# Histone lactylation encodes innate immune memory in atherosclerosis: a hypothesis of epigenetic bookmarking for residual inflammatory risk

**DOI:** 10.1186/s13148-026-02224-8

**Published:** 2026-08-12

**Authors:** Haobo Yang, Yonghui Zhang, Yunfeng Yu, Jiale Zhu, Liping Wang, Weixiong Jian

**Affiliations:** 1 Hunan University of Chinese Medicine, Changsha, 410208 China; 2 Hunan Provincial Key Laboratory of TCM Diagnostics, Institute of National Key Discipline in TCM Diagnostics, Changsha, 410208 China; 3 School of Traditional Chinese Medicine, Hunan University of Medicine, Huaihua, 418000 China

**Keywords:** Histone lactylation, H3K18 lactylation, Trained immunity, Atherosclerosis, Coronary heart disease, Immunometabolism, HIF-1alpha, Residual inflammatory risk

## Abstract

Histone lactylation, particularly H3K18 lactylation (H3K18la), has emerged as a lactate-derived epigenetic modification that links metabolic reprogramming to sustained chromatin states. Here, we advance a hypothesis that the lactate-p300-H3K18la axis functions as an epigenetic memory code, converting transient glycolytic signals into persistent transcriptional responsiveness in innate immune cells—a mechanism we term “context-dependent lactylation memory.” We integrate evidence from three converging domains: the persistence of lactylation at trained immune loci, the requirement of H3K18la for trained immunity demonstrated in recent BCG vaccination studies, and the hypoxic, glycolytic microenvironment of the atherosclerotic plaque. We propose that cardiovascular risk factors stabilize HIF-1α, which drives glycolytic reprogramming and sustained lactate production. Lactate-derived lactyl-CoA then serves as a substrate for p300/CBP-mediated H3K18la deposition at immune gene regulatory elements, creating a poised chromatin state that sustains innate immune hyperresponsiveness. This cascade provides a mechanistic explanation for residual inflammatory risk, the observation that elevated cardiovascular events persist despite optimal lipid-lowering therapy. The framework is constrained by the context-dependent duality of lactylation, whereby cell type, lysine site, and metabolic flux direction jointly determine whether the modification drives pro-inflammatory or reparative outcomes. We delineate testable predictions that define a roadmap for empirical validation or refutation of the lactate–lactylation memory hypothesis in human CHD.

## Introduction

Coronary heart disease (CHD) remains the leading cause of mortality worldwide, with atherosclerosis as its pathological substrate. Although lipid-lowering therapies and revascularization procedures have substantially improved clinical outcomes, a persistent challenge remains: even among patients achieving optimal lipid control, residual inflammatory risk continues to drive disease progression and precipitate acute cardiovascular events [1, 2]. The CANTOS trial validated the inflammation hypothesis by demonstrating that targeting interleukin-1β reduces cardiovascular events independently of lipid lowering [3, 4]. Yet a fundamental mechanistic question persists—what sustains inflammatory memory long after the inciting metabolic insult has subsided? This article is presented as a hypothesis paper; no original experimental data are reported. We synthesize evidence from published literature to formulate a falsifiable mechanistic framework, delineate its constraints, and specify the experimental predictions by which it can be tested. Throughout the manuscript, 'our hypothesis' refers to this proposed framework and its corollaries.

The pathogenesis of CHD is driven by the retention and oxidation of apolipoprotein B-containing lipoproteins in the arterial intima, which triggers endothelial activation and the recruitment of circulating monocytes via chemokine gradients involving the CCL2/CCR2 axis [2, 5]. Infiltrating monocytes differentiate into macrophages, internalize modified lipoproteins via scavenger receptors including CD36 and SR-A, and form lipid-laden foam cells that constitute the earliest atherosclerotic lesions [6]. As plaques progress, a necrotic core develops from apoptotic foam cells combined with defective efferocytosis, while sustained inflammatory signaling perpetuates monocyte recruitment [7]. Monocyte-derived macrophages within the plaque microenvironment display remarkable functional heterogeneity, ranging from pro-inflammatory populations enriched at the rupture-prone plaque shoulder to lipid-handling TREM2hi populations associated with the necrotic core and reparative populations associated with fibrosis [8]. Two distinct anatomical compartments contribute to this maladaptive immune response. Central trained immunity originates in bone marrow hematopoietic stem and progenitor cells (HSPCs), where cardiovascular risk factors including hyperglycemia, hyperlipidemia, and smoking induce lasting epigenetic reprogramming that biases myelopoiesis toward a hyperinflammatory monocyte phenotype [7, 9, 10]. Peripheral trained immunity operates within the plaque itself, where tissue-resident and monocyte-derived macrophages undergo local metabolic and epigenetic reprogramming in response to oxLDL, hypoxia, and cytokines, sustaining a self-amplifying inflammatory circuit [7, 11]. This dual compartment model explains why systemic risk factors can drive plaque progression even after lipid normalization, and it provides the pathophysiological context for our hypothesis that histone lactylation encodes durable innate immune memory linking metabolic dysregulation to residual inflammatory risk.

**Fig. 1:**
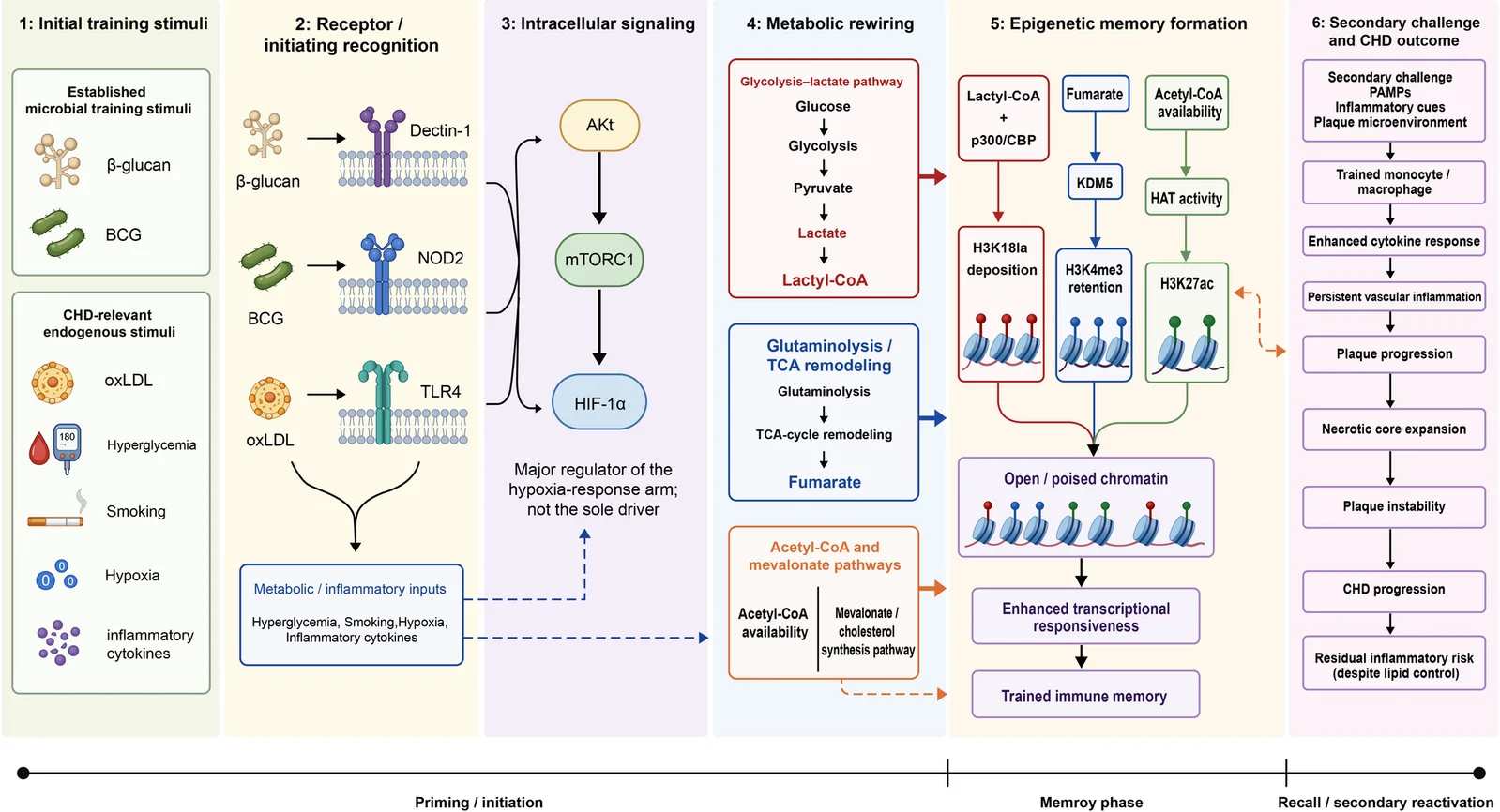
Integrated mechanistic framework linking trained-immunity initiation to epigenetic memory and coronary heart disease progression. Established microbial training stimuli, including β-glucan and Bacillus Calmette–Guérin (BCG), and coronary heart disease (CHD)-relevant endogenous stimuli, including oxidized low-density lipoprotein (oxLDL), hyperglycemia, smoking, hypoxia, and inflammatory cytokines, initiate receptor-mediated or metabolic/inflammatory signaling. β-Glucan–Dectin-1, BCG–NOD2, and oxLDL–TLR4 signaling converge on Akt/mTORC1 and HIF-1α, while additional CHD-related inputs act through parallel pathways. These signals induce three interconnected metabolic programs: glycolysis and lactate production, glutaminolysis/TCA-cycle remodeling with fumarate accumulation, and acetyl-CoA/mevalonate pathway activation. The resulting metabolites support H3K18 lactylation (H3K18la) through the lactyl-CoA–p300/CBP axis, H3K4me3 retention through fumarate-mediated KDM5 inhibition, and H3K27ac deposition through acetyl-CoA-dependent histone acetyltransferase activity. Together, these modifications establish an open or poised chromatin state associated with enhanced transcriptional responsiveness and trained immune memory. Upon secondary challenge, trained monocytes and macrophages may mount amplified cytokine responses, thereby sustaining vascular inflammation, plaque progression, necrotic-core expansion, plaque instability, and residual inflammatory risk despite lipid control. The proposed extension from H3K18la deposition to durable CHD-associated innate immune memory remains to be directly validated. Solid arrows summarize the principal relationships depicted, whereas dashed arrows indicate indirect or parallel contributory inputs

The concept of trained immunity provides a compelling mechanistic framework. Trained immunity describes the capacity of innate immune cells—including monocytes, macrophages, natural killer cells, and hematopoietic stem and progenitor cells (HSPCs)—to acquire a long-term hyperresponsive phenotype following exposure to specific stimuli [6, 12]. At the molecular level, trained immunity is initiated when defined ligands engage pattern recognition receptors: β-glucan signals through Dectin-1, oxLDL through toll-like receptor 4 (TLR4), and Bacillus Calmette-Guérin (BCG) through nucleotide-binding oligomerization domain 2 (NOD2) [12, 13]. Receptor engagement activates the Akt/mTOR/HIF-1α signaling axis, which in turn drives a coordinated metabolic shift characterized by three parallel rewiring events: increased aerobic glycolysis with elevated glucose consumption and lactate production, glutaminolysis with fumarate accumulation, and upregulation of the mevalonate pathway for cholesterol synthesis [13–16]. These metabolic intermediates serve as cofactors and substrates for epigenetic regulators: fumarate inhibits the KDM5 histone demethylase to sustain H3K4me3 at proinflammatory gene promoters, mevalonate-derived acetyl-CoA fuels H3K27ac deposition, and lactate-derived lactyl-CoA drives p300/CBP-mediated H3K18la deposition—each contributing to a durable open chromatin state at immune effector loci [14, 15, 17]. Unlike adaptive immunity, trained immunity is mediated by this metabolic–epigenetic coupling, enabling functional alterations that persist for months to years [13]. In CHD, risk factors such as hyperglycemia, oxLDL, and smoking function as training stimuli, activating these same pathways to induce a pre-activated state in bone marrow progenitors and circulating monocytes [9]. Trained cells recruited to the vessel wall release amplified proinflammatory cytokines, promoting endothelial dysfunction and plaque destabilization [5, 10]. Myocardial infarction itself can serve as a training stimulus via damage-associated molecular patterns, establishing a vicious inflammatory cycle [18]. Figure 1 has been revised to illustrate these ligand–receptor–signaling–metabolism–epigenetic initiating events.

Concurrently, the discovery of histone lactylation by Zhang and colleagues (Nature, 2019) redefined lactate as an epigenetic substrate [19]. Lactate-derived lactyl-CoA donates lactyl groups to histone lysine residues, most notably H3K18. Zhang et al. proposed a “lactate clock” whereby lactate drives a temporal shift in macrophage polarization through epigenetic mechanisms [19]. These two trajectories converged in 2025 with the landmark study by Ziogas and colleagues (Cell), demonstrating that BCG-trained monocytes exhibit H3K18la enrichment at regulatory elements, that H3K18la persists for at least 90 days post-stimulus, and that LDHA and EP300 polymorphisms modulate trained immunity phenotypes. Pharmacological inhibition of lactate production or lactylation blocked trained immunity, establishing causality [17].

However, Ziogas et al. employed an exogenous microbial stimulus (BCG) in healthy volunteers. Whether the lactate–lactylation axis similarly drives CHD-associated inflammation under endogenous metabolic stress, and whether this axis accounts for residual inflammatory risk, constitutes a critical knowledge gap. Here, we advance a hypothesis with three integrated components: (i) the kinetic properties of histone lactylation—particularly its persistence relative to acetylation—enable it to function as a durable epigenetic memory trace; (ii) in the hypoxic, glycolytic atherosclerotic plaque microenvironment, sustained HIF-1α-driven lactate production promotes chronic H3K18la deposition at immune gene loci, encoding a “context-dependent lactylation memory” that maintains chromatin in a poised state decoded by local microenvironmental cues; and (iii) this epigenetic memory provides a mechanistic basis for residual inflammatory risk that is experimentally testable through specific predictions and can inform the development of immunometabolic memory-targeting therapeutic strategies. The timeline of these integrated processes is illustrated in Fig. 1.

## Histone lactylation: durable persistence enables epigenetic memory

### Writers, erasers, and readers of the lactylation code

Lactate functions as an energy substrate [20–24], a GPR81 (HCAR1) receptor ligand, and an epigenetic regulator via histone lactylation [25–27]. Its conversion to an epigenetic signal requires metabolic activation to lactyl-CoA [28–30]. Three enzyme classes orchestrate this dynamic system (Table 1). The writers—principally p300 and CBP—catalyze lactyl transfer from lactyl-CoA to histone lysines, with H3K18la being the most extensively characterized modification [17, 28]. p300/CBP activity responds directly to intracellular lactate concentration, providing the mechanistic basis for coupling glycolytic flux to epigenetic output. Beyond histones, AARS1 and AARS2 catalyze non-histone lactylation via a lactyl-AMP intermediate [31]. The erasers comprise Class I HDACs (HDAC1–3) operating via a zinc-dependent mechanism, and sirtuins (SIRT1–3) catalyzing NAD^+^-dependent delactylation [32–34]. The energy-sensing property of sirtuins couples delactylation to cellular redox state—a relationship of particular relevance in the hypoxic plaque microenvironment. The readers include bromodomain- and YEATS domain-containing proteins that recognize lactylated lysines and recruit chromatin remodeling complexes such as SWI/SNF [35, 36].

**Table 1:** Histone lactylation enzymatic machinery and CHD significance

Functional class	Representative enzyme	Catalytic/recognition mechanism	Significance in CHD context
Writer	p300/CBP	Lactyl-CoA-dependent histone lactylation	Upregulated in hypoxia; drives proinflammatory or reparative programs
Writer	AARS1/2	Lactyl-AMP-mediated non-histone lactylation	Regulates metabolic enzyme activity; affects cellular energetics
Eraser	HDAC1–3	Zinc-dependent delactylation	Activity potentially suppressed in inflammatory microenvironment
Eraser	SIRT1–3	NAD^+^-dependent delactylation	Energy-sensing mechanism; functionally constrained under hypoxia
Reader	BRG1/DPF2	Recognizes lactylated lysine; recruits SWI/SNF complex	Mediates transcriptional activation of reparative genes

Lactyl-CoA, the obligate substrate for enzymatic histone lactylation, is synthesized from lactate and coenzyme A by two nuclear enzymes. ACSS2 converts lactate to lactyl-CoA and couples with the histone lactyltransferase KAT2A, while GTPSCS couples with p300/CBP to channel lactyl-CoA directly into H3K18la deposition [28, 29]. Both enzymes are primarily regulated by intracellular lactate availability rather than by transcriptional induction, establishing a direct link between glycolytic flux and histone lactylation. CHD risk factors converge on elevating intracellular lactate through distinct mechanisms. Hyperglycemia drives glycolytic flux by mass action. OxLDL engages TLR4 to activate the mTOR/HIF-1α axis, upregulating HK2 and LDHA [15, 16]. Plaque hypoxia stabilizes HIF-1α, transcriptionally amplifying GLUT1, PDK1, and LDHA expression [37, 38]. The net effect is a substrate push: elevated glycolytic flux increases lactate availability, which increases lactyl-CoA production, which increases H3K18la deposition.

The enzymes that remove histone lactylation are also subject to regulation by CHD risk factors. HDAC1–3 and SIRT1/SIRT3 function as histone lysine delactylases [33, 34]. SIRT1 expression or activity has been reported to decline in several atherogenic and metabolic-stress settings, although its regulation in specific human monocyte subsets and its direct impact on histone delactylation in CHD remain insufficiently defined. This downregulation would be predicted to impair delactylation capacity, favoring net H3K18la accumulation at immune gene loci. HDAC1–3 expression changes in response to CHD risk factors have not been systematically assessed with respect to their delactylase function and represent a knowledge gap.

### Why lactylation outlasts acute metabolic signals

**Fig. 2:**
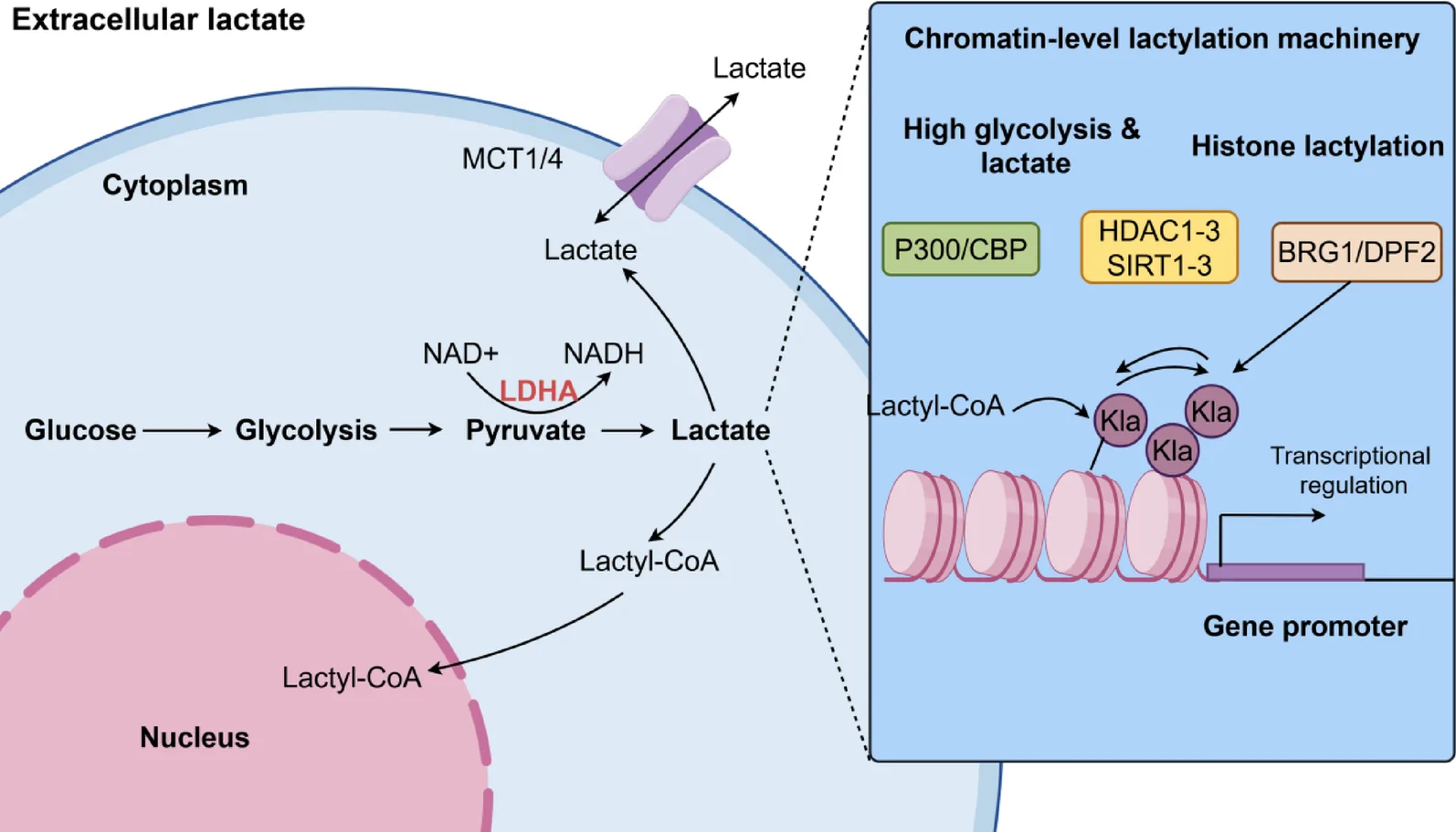
Molecular mechanism of chromatin-level histone lactylation encoding epigenetic memory. **A** The enzymatic machinery of histone lactylation: writers (p300/CBP catalyzing lactyl-CoA-dependent H3K18la; AARS1/2 mediating non-histone lactylation via lactyl-AMP), erasers (HDAC1–3 catalyzing zinc-dependent delactylation; SIRT1–3 catalyzing NAD^+^-dependent delactylation), and readers (bromodomain- and YEATS domain-containing proteins including BRG1/DPF2 that recruit SWI/SNF chromatin remodeling complexes). **B** Basis of lactylation memory durability: the additional hydroxyl group on the lactyl moiety reduces eraser catalytic efficiency relative to acetylation, while compartmentalized lactyl-CoA metabolism creates a threshold effect that sustains marks during metabolic quiescence. **C** The HIF-1α–lactate–lactylation feedforward loop: plaque hypoxia stabilizes HIF-1α, upregulating glycolytic enzymes (GLUT1, HK2, LDHA, PDK1) that drive sustained lactate production, providing substrate for chronic H3K18la deposition

A defining feature relevant to our hypothesis is the sustained locus-level enrichment of H3K18la observed in specific trained-immunity contexts. This locus-level persistence provides a rationale for investigating its potential role in durable epigenetic memory. However, Ziogas et al. demonstrated that H3K18la enrichment at trained immune gene promoters remains detectable at the cell population level for at least 90 days following BCG vaccination [17]. This locus-level persistence—whether maintained by sustained writer activity, inefficient eraser turnover, or self-reinforcing chromatin states—distinguishes lactylation from more transient modifications and provides the kinetic basis for its proposed role in encoding durable epigenetic memory. Three mechanistic factors likely contribute. First, the additional hydroxyl group on the lactyl moiety reduces eraser catalytic efficiency: HDAC1–3 delactylation rates are significantly lower than deacetylation rates in vitro [28]. Second, lactyl-CoA metabolism is subcellularly compartmentalized. Nuclear enzymes including GTPSCS and ACSS2 function as dedicated lactyl-CoA synthetases, generating a nuclear lactyl-CoA pool that is physically coupled to histone lactyltransferase complexes such as p300/CBP and KAT2A [29, 39]. Lactyl-CoA is present at low basal concentrations (approximately 0.011 pmol/10^6^ cells, roughly 30–350-fold lower than acetyl-CoA and succinyl-CoA) and rises substantially only under conditions of elevated glycolytic flux [30]. This limited basal availability may create a threshold effect: once H3K18la is deposited during a glycolytic burst, the mark may persist during subsequent metabolic quiescence because basal lactyl-CoA concentrations are insufficient to sustain continuous writer activity, while HDAC1–3-mediated delactylation remains kinetically constrained relative to deacetylation [33]. The subcellular compartmentalization of acyl-CoA metabolism and its implications for chromatin regulation have been comprehensively reviewed [39, 40]. Third, reader protein recruitment and chromatin remodeling at lactylated loci may establish self-reinforcing chromatin states resistant to passive erasure. BRG1 (SMARCA4), the ATPase subunit of the SWI/SNF chromatin remodeling complex, has been identified as a specific reader of H3K18la, forming more hydrogen bonds with K18-lactylated H3 than with unmodified H3 and facilitating chromatin accessibility at target gene promoters [35]. DPF2, another SWI/SNF-associated protein, was independently identified as a histone lactylation reader that drives transcription and tumorigenesis [36]. These reader proteins, together with the bromodomain- and YEATS domain-containing proteins discussed in Fig. 2, may stabilize lactylated chromatin states through self-reinforcing remodeling. We note that histone lactylation is not the only epigenetic mark with demonstrated durability in trained immunity. H3K4me3 at promoters of trained genes persists through fumarate-mediated inhibition of KDM5 histone demethylases [14], and H3K27ac at enhancer regions is maintained via mevalonate pathway-driven acetyl-CoA production [15, 41]. The distinctive contribution of lactylation lies in its direct dependence on glycolytic flux through lactate availability and lactyl-CoA synthesis, positioning it as a metabolic sensor that complements these parallel durable marks.

This persistence enables lactylation to serve as a memory trace, recording past episodes of glycolytic activation. Critically, we distinguish between structural maintenance of the mark (memory retention) and functional transcriptional output (memory expression). As elaborated below, the same H3K18la mark can scaffold either proinflammatory or reparative gene programs, with the local microenvironment acting as the decoder. In trained immunity, this explains how BCG vaccination primes monocytes 90 days later [17]. In CHD, we hypothesize that sustained exposure to the pro-glycolytic plaque microenvironment establishes durable lactylation landscapes that perpetuate inflammatory gene expression during metabolic quiescence. However, lactylation persistence is not universal. Raychaudhuri et al. demonstrated that H3K18la and H3K9la in activated human CD8 + T cells begin to decline within 48 h of stimulation in vitro, indicating that lactylation turnover is highly context-dependent and may be more transient in rapidly proliferating lymphoid cells than in long-lived innate immune populations [42]. Our hypothesis therefore predicts that lactylation durability is greatest in cell types with low proliferative turnover and sustained glycolytic metabolism, such as tissue-resident macrophages and HSPCs, rather than being a general property of all lactylated loci in all cell types. The molecular machinery underlying this persistent lactylation is depicted in Fig. 2.

### The HIF-1α–lactate–lactylation axis as the upstream driver in CHD

We propose HIF-1α as the master transcriptional regulator linking the plaque microenvironment to sustained lactate production [37, 38]. Atherosclerotic plaques exhibit regional hypoxia driven by increased oxygen diffusion distances, high metabolic demand of inflammatory cells, and impaired neovascularization [43]. Under hypoxia, PHD-mediated HIF-1α hydroxylation is suppressed, allowing HIF-1α stabilization, nuclear translocation, and HRE binding [44–46]. Plaque oxidative stress amplifies HIF-1α signaling by inhibiting PHD activity [47, 48], and IRE1 RNase can stabilize HIF-1α independently of XBP1 splicing [49]. Activated HIF-1α upregulates GLUT1, HK2, PFK1, LDHA, and PDK1, shunting pyruvate toward lactate production [50–52]. In oxLDL-stimulated macrophages, HIF-1α upregulation accompanies marked lactate accumulation; HIF-1α inhibition in post-MI models reduces glycolytic enzyme expression and improves myocardial energetics [53]. We propose that cardiovascular risk factors promote HIF-1α stabilization and glycolytic reprogramming, leading to sustained lactate production, increased H3K18 lactylation, a trained-immunity-like epigenetic state, persistent inflammation, and ultimately plaque progression. This cascade identifies multiple testable nodes for therapeutic intervention. We note that HIF-1α, while central to the hypoxia-driven arm of this cascade, does not act in isolation. Additional pathways converge on glycolytic reprogramming in the plaque microenvironment independently of or synergistically with HIF-1α: mTORC1 is activated by oxLDL via TLR4 signaling, inflammasome-derived IL-1β amplifies glycolytic gene expression, and the cholesterol biosynthesis pathway generates mevalonate-derived metabolites that further drive trained immunity [15, 16]. Our designation of HIF-1α as the “master transcriptional regulator” is therefore specific to the hypoxia response arm of this mechanism and does not imply that HIF-1α is the sole driver of glycolytic flux in CHD.

### Context-dependent duality of lactylation: a constraint on the hypothesis

A crucial constraint on our hypothesis is the striking context-dependence of lactylation’s biological effects [54]. Lactylation is not uniformly proinflammatory; it exerts opposing consequences depending on cell type, lysine residue, and metabolic flux direction.

*Cell-type specificity* In macrophages, MCT4 deficiency impairs lactate efflux, causing intracellular lactate retention that drives H3K18la at reparative gene loci (Arg1, Il10), conferring atheroprotection [55, 56]. Conversely, EP300-mediated H3K18la upregulates METTL3, accelerating m^6^A-dependent SLC7A11 mRNA degradation and promoting macrophage ferroptosis and atherosclerosis [57, 58]. In endothelial cells, ASF1A/p300-mediated H3K18la promotes SNAI1 transcription and EndMT [59], whereas exercise-induced MeCP2 K271 lactylation represses EREG, exerting atheroprotection [60]. In VSMCs, Glut1-dependent H3K18la and TRAP1-facilitated H4K12 lactylation drive senescence [61–63]; NR4A3-mediated lactylation participates in medial calcification [64].

*Site-specific divergence (referring to distinct histone lysine residues)* SLC22A6-dependent H3K9la aggravates endothelial dysfunction via SCD1 activation, whereas H3K18la—as discussed above—drives context-dependent outcomes ranging from reparative gene activation to ferroptosis depending on cell type and metabolic flux. H3K9la or SCD1 inhibition alleviates endothelial dysfunction and atherosclerosis [65]. The molecular basis for site-specificity—likely involving differential reader recruitment, local chromatin context, or combinatorial modification crosstalk—remains poorly characterized.

*Flux directionality* MCT4 deficiency induces a reparative H3K18la landscape by blocking lactate export [56]. Conversely, efficient export limits intracellular substrate while elevating extracellular lactate available for GPR81 signaling or MCT1-mediated import by neighboring cells. We propose that highly glycolytic plaque macrophages function as “lactate donors,” with exogenous lactate imported by oxidative “receiver” cells (fibroblasts, VSMCs) via the MCT1/MCT4 shuttle [23], establishing H3K18la in bystander cells and propagating epigenetic states across the plaque in a paracrine-like manner.

**Fig. 3:**
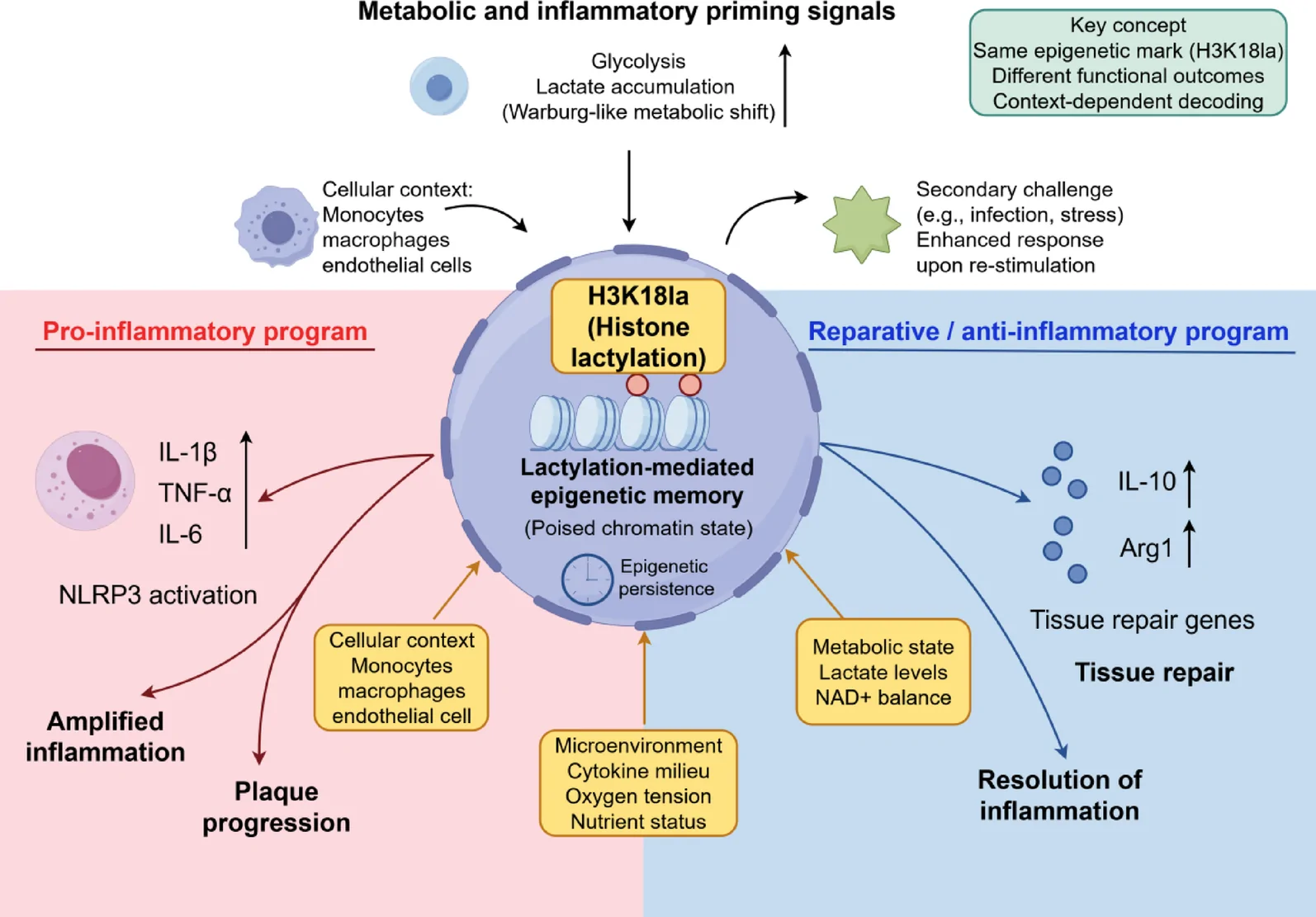
Cellular mechanisms of trained immunity in monocytes/macrophages mediated by the lactate–p300–H3K18la axis. **A** Training phase: atherogenic stimuli (β-glucan, oxLDL, hyperglycemia) induce metabolic reprogramming toward aerobic glycolysis, glutaminolysis, and cholesterol synthesis, converging on p300/CBP-mediated H3K18la deposition at promoters and enhancers of proinflammatory genes (IL6, TNF, NLRP3). **B** Memory phase: H3K18la persists at trained loci for at least 90 days, maintaining chromatin in a poised state characterized by increased accessibility and latent transcriptional potential. Parallel epigenetic pathways (H3K27ac via mevalonate, H3K4me3 via fumarate-mediated KDM5 inhibition) cooperate with H3K18la. **C** Context-dependent decoding: upon restimulation, the same H3K18la mark is decoded by the local microenvironment. In proinflammatory contexts (EP300 overexpression, oxLDL-rich), it drives ferroptosis (via METTL3/m6A/SLC7A11) and EndMT (via SNAI1); in reparative contexts (MCT4 deficiency, exercise), it drives M2 polarization (Arg1, Il10) and atheroprotection. **D** Non-enzymatic contribution: S-lactoylglutathione (LGSH) directly lactylates histone lysines independently of p300, predicted to predominate in the severely hypoxic plaque core

We therefore conceptualize lactylation not as a binary pro- or anti-inflammatory signal, but as a metabolic–epigenetic switch decoded by cell-intrinsic and microenvironmental factors. Throughout this hypothesis article, we use the term 'context-dependent lactylation memory' to refer specifically to three molecular dimensions that jointly determine the functional output of histone lactylation: (1) cell-type specificity—the same modification (e.g., H3K18la) can drive pro-inflammatory programs in macrophages via the p300/CBP axis while promoting reparative programs in MCT4-deficient macrophages; (2) site-specific divergence—H3K18la and H3K9la engage distinct downstream transcriptional programs through different writers and readers; and (3) lactate flux directionality—whether lactate is retained intracellularly (promoting histone lactylation) or exported (limiting substrate availability while activating GPR81 signaling). This duality represents a critical falsifiability constraint on our hypothesis: if H3K18la uniformly drives either protective or pathogenic programs, the context-dependent memory model would be refuted. A detailed cellular cascade of this context-dependent process is shown in Fig. 3.

## H3K18la as an epigenetic bookmark of trained immunity

### Metabolic and epigenetic foundations

Trained immunity in atherosclerosis is increasingly recognized as a central mechanism linking metabolic dysregulation to sustained vascular inflammation, and has been proposed to recast atherosclerosis as an immune-metabolic memory disorder [66]. The process is initiated when defined ligands, including β-glucan, BCG, and atherogenic factors such as oxLDL, engage pattern recognition receptors (Dectin-1, NOD2, TLR4) on innate immune cells, triggering a coordinated metabolic shift with three interconnected components: increased aerobic glycolysis with elevated glucose consumption and lactate production, enhanced glutamine metabolism with fumarate accumulation, and upregulation of the mevalonate pathway for cholesterol synthesis [15, 16].

Each of these metabolic adaptations generates metabolites that function as obligatory substrates or cofactors for specific epigenetic enzymes. Fumarate, accumulating through glutaminolysis and TCA cycle remodeling, competitively inhibits the KDM5 family of H3K4me3 demethylases, thereby sustaining H3K4me3 at promoters of proinflammatory genes including IL6 and TNF in β-glucan-trained macrophages [14, 67]. Concurrently, glycolysis-derived and mevalonate-derived acetyl-CoA drives p300/CBP-mediated H3K27ac deposition at immune gene enhancers, establishing a permissive chromatin landscape [15]. The mTOR and HIF-1α pathways integrate nutrient and oxygen sensing to coordinate this metabolic–epigenetic reprogramming [16]. At the chromatin level, the trained phenotype is characterized by sustained H3K27ac and H3K4me3 at enhancers and promoters of proinflammatory and metabolic genes, increased chromatin accessibility, and latent transcriptional potential that enables amplified cytokine responses upon restimulation [68, 69]. These marks are enzymatically deposited and removed, and are diluted through cell division, accounting for the durable yet ultimately reversible nature of trained immunity, which typically persists for months to years rather than for the lifetime of the organism [13, 70, 71].

A key question raised by this established framework is how a transient metabolic signal is converted into a chromatin state that outlasts the initiating stimulus by weeks to months. H3K27ac has a molecular half-life of minutes to hours, and H3K4me3 retention depends on sustained fumarate-mediated KDM5 inhibition. Neither mechanism alone fully explains the durability of trained immunity in slowly dividing or quiescent cells such as tissue-resident macrophages and HSPCs. Histone lactylation, which can display sustained locus-level enrichment in specific trained-immunity contexts, may serve as a complementary memory-associated mark that helps bridge this mechanistic gap.

### The lactate–p300–H3K18la axis in innate immune memory

The Ziogas et al. (2025) study provides the strongest evidence for H3K18la as a bona fide epigenetic mark of innate immune memory [17]. Key findings: (i) lactate release from PBMCs positively correlated with cytokine production capacity at 90 days post-BCG; (ii) H3K18la was enriched at open chromatin and active enhancers, correlating with active transcription; (iii) H3K18la persisted in vivo 90 days post-vaccination; (iv) LDHA and EP300 polymorphisms correlated with individual variation in trained cytokine responses—human genetic evidence for a causal link; and (v) pharmacological inhibition of LDHA (GSK2837808A) or p300 (A-485) blocked trained immunity, establishing causality.

It is important to note that BCG vaccination is epidemiologically associated with reduced, rather than increased, cardiovascular mortality [72], likely reflecting the net survival benefit of trained immunity against heterologous infections. Our hypothesis therefore does not propose that BCG promotes CHD; rather, it proposes that the H3K18la-dependent epigenetic machinery characterized in the BCG model can be co-opted by endogenous atherogenic stimuli to encode maladaptive innate immune memory in the CHD plaque microenvironment.

Trained immunity is multifactorial. Parallel pathways include fumarate-mediated KDM5 inhibition and H3K4me3 retention [14], and cholesterol synthesis-driven H3K27ac deposition [15, 41]. Lactylation is thus a critical node within a broader regulatory network. An important consideration that warrants attention is monocyte heterogeneity. Human blood monocytes comprise three functionally distinct subsets defined by CD14 and CD16 expression: classical monocytes (CD14 +  + CD16−), which constitute approximately 85% of circulating monocytes and are primarily phagocytic and recruited to inflammatory sites; intermediate monocytes (CD14 +  + CD16 +), which exhibit high antigen presentation capacity, proinflammatory cytokine production, and have been independently associated with coronary artery disease severity; and non-classical monocytes (CD14 + CD16 + +), which patrol the endothelium and contribute to tissue repair and resolution [6, 13]. A recent single-cell high-dimensional analysis identified unique intermediate monocyte subsets associated with sex differences in coronary artery disease, further underscoring the functional granularity of monocyte populations in cardiovascular contexts [73]. Whether histone lactylation and trained immune memory operate uniformly across monocyte subsets is currently unknown and represents a critical open question. Given that classical and intermediate monocytes display higher glycolytic flux relative to non-classical monocytes, and that glycolytic rate is a primary determinant of intracellular lactate availability, we speculate that classical and intermediate monocytes may be more permissive for H3K18la deposition and trained immunity establishment. Conversely, non-classical monocytes, which rely more on oxidative phosphorylation, may be less susceptible to lactate-driven epigenetic reprogramming. This subset-specific vulnerability, if experimentally confirmed, would have implications for therapeutic targeting: strategies aimed at erasing lactylation memory may need to prioritize the classical and intermediate compartments rather than targeting all monocytes uniformly.

Furthermore, a dual enzymatic/non-enzymatic system operates. Beyond p300/CBP-mediated transfer of L-lactyl-CoA to histone lysines, which generates L-lactylation (KL-la)—the predominant form detected on mammalian histones—S-D-lactoylglutathione (LGSH), a glyoxalase intermediate, can directly transfer D-lactyl groups to histone lysines independently of acetyltransferases, producing D-lactylation (KD-la) [65, 66]. Whether D-lactylation contributes meaningfully to the histone lactylation landscape in the plaque microenvironment is currently unknown. Given that L-lactylation, not D-lactylation, is the major form identified in mammalian cells, the non-enzymatic LGSH pathway should be regarded as a potential supplementary mechanism that requires direct experimental validation in cardiovascular tissues before its significance can be assessed.

## The plaque as a lactylation memory hub: hypothesis and evidence

### Central hypothesis: the atherosclerotic plaque encodes and propagates lactylation memory

We advance the hypothesis that the atherosclerotic plaque functions as a “lactylation memory hub”—a microenvironment in which sustained HIF-1α-driven lactate production chronically deposits H3K18la at immune gene regulatory elements, establishing an epigenetic memory that perpetuates inflammation independently of circulating lipid levels. This hypothesis rests on three premises: (i) CHD-relevant risk factors (oxLDL, hyperglycemia, hypoxia) activate the HIF-1α–LDHA–lactate axis in a manner analogous to BCG [9, 37]; (ii) these chronic stimuli induce H3K18la with persistence sufficient to outlast the initiating metabolic signal; and (iii) the resulting chromatin state predisposes cells to amplified inflammatory responses upon secondary stimulation.

We acknowledge that only the first premise currently enjoys strong experimental support—oxLDL, hyperglycemia, and hypoxia all stabilize HIF-1α and promote glycolysis [74, 75]. The second and third premises remain directly untested. No study has demonstrated that oxLDL or hyperglycemic exposure establishes an H3K18la memory trace persisting weeks to months after stimulus removal.

The plaque microenvironment nonetheless provides the necessary biochemical conditions: regional hypoxia, hyperglycemia, oxLDL accumulation, and chronic inflammatory infiltrate [74–76]. Indirect evidence supports the plausibility of this thesis, and we distinguish two categories of supportive data. The first category demonstrates that histone lactylation contributes to atherosclerosis progression: MCT4 deficiency-induced H3K18la protects against atherosclerosis [56], EP300-mediated H3K18la promotes ferroptosis and disease progression [58], and H3K9la exacerbates endothelial dysfunction and atherosclerosis [65]. However, these studies establish lactylation as a disease modifier, not as a mediator of innate immune memory. The second category comprises the Ziogas et al. BCG vaccination study [17], which demonstrated that H3K18la persists for at least 90 days and is required for trained immunity, and provides the only direct evidence to date linking lactylation to epigenetic memory. Our hypothesis bridges these two categories by proposing that the disease-modifying lactylation events documented in atherosclerosis models are sustained by the same memory-encoding mechanisms characterized in the BCG system. This bridging inference, while mechanistically plausible, remains to be experimentally validated. The critical experiment—demonstrating that lactylation marks established during a transient metabolic insult persist and influence subsequent inflammatory responses in the absence of ongoing stimulation—has not been performed. This represents the most direct test of our hypothesis.

### Central and peripheral lactylation memory

The trained immunity framework distinguishes between central memory (bone marrow HSPCs) and peripheral memory (tissue-resident immune cells) [77, 78]. We predict that both compartments contribute to lactylation-mediated memory in CHD. Cardiovascular risk factors access the bone marrow niche and reprogram HSPCs toward enhanced myelopoiesis and proinflammatory differentiation [17, 77]; even transient IL-1β exposure durably reprograms human HSPCs [10]. Whether lactylation participates in this central memory is unknown. Given lactate’s role in HSC maintenance [79, 80], we predict that HSPC lactylation marks established during metabolic stress persist through HSPC self-renewal and are propagated to differentiated myeloid progeny.

Peripherally, we hypothesize a self-amplifying loop: trained monocytes infiltrating the arterial wall encounter hypoxia, oxLDL, and cytokines that reinforce glycolytic programming, leading to sustained lactate production and H3K18la deposition, which in turn further sensitizes cells to stimulation. Non-immune vascular cells may also acquire lactylation memory [81]. Post-MI, DAMPs further train HSPCs and plaque-resident cells, establishing injury memory that perpetuates inflammation [18]. We predict that H3K18la levels in circulating monocytes will be elevated post-MI and will correlate with recurrent event risk.

Whether endogenous CHD risk factors such as hyperglycemia, smoking, or hypertension can trigger H3K18la in circulating monocytes independently of atherosclerotic plaque burden has not been directly tested. The BCG vaccination study demonstrates the principle that a systemic stimulus unrelated to atherosclerosis can induce durable H3K18la in human PBMCs [17], suggesting that this possibility merits investigation.

## Therapeutic implications of the lactylation memory hypothesis

If our hypothesis is correct—that durable H3K18la deposition encodes immunometabolic memory in CHD—then therapeutic strategies must distinguish between preventing memory establishment and erasing consolidated marks. We propose that this distinction carries direct consequences for drug development. We use the term “lactylation memory” to refer specifically to the structural maintenance of H3K18la at trained gene loci beyond the duration of the initiating metabolic stimulus, creating a poised chromatin state that persists during metabolic quiescence. This is distinct from “memory expression,” which refers to the functional transcriptional output of that poised state upon restimulation. Whether continued p300/CBP activity is required to maintain lactylation memory is currently an open question with direct therapeutic relevance. Two competing models are plausible. In an active maintenance model, sustained writer activity continuously re-deposits H3K18la to counteract basal HDAC1–3-mediated delactylation, implying that p300 or LDHA inhibition would lead to memory erasure. In a passive persistence model, the slow kinetics of delactylation combined with reader protein stabilization of chromatin structure allow the mark to persist without continuous re-writing, implying that p300 or LDHA inhibition would prevent new memory formation but would not erase existing marks. The principal targets within this axis are summarized in Table 2.

**Table 2:** Therapeutic targets in the lactate–lactylation–trained immunity axis

Target	Intervention	Rationale	Evidence Level
p300/CBP	A-485 (inhibitor)	Blocks deposition of H3K18la and other acylations	Validated in BCG-trained human monocytes [17]; not yet tested in atherosclerosis models
MCT4	Genetic deletion / Inhibition	Promotes intracellular lactate retention, inducing reparative H3K18la landscape	Validated in murine atherosclerosis models (atheroprotective) [56]
LDHA	GSK2837808A (inhibitor)	Reduces lactate production, limiting substrate for lactylation	Validated in BCG-trained human monocytes [17]; not yet tested in atherosclerosis models
SIRT1	Resveratrol / SRT1720 (activators)	Enhances NAD^+^-dependent delactylation, potentially erasing lactylation memory	Validated in general CVD models; direct evidence for memory erasure lacking
HIF-1α	PX-478 (inhibitor)	Suppresses glycolytic reprogramming upstream of lactate production	Validated in murine atherosclerosis models (reduces plaque progression)

Note: MCT4 inhibition and HIF-1α inhibition are both atheroprotective despite opposing effects on intracellular lactate. MCT4 blockade retains intracellular lactate, directing H3K18la to reparative gene promoters (Arg1, Il10) [55]; HIF-1α inhibition reduces glycolytic flux and lactate availability, and would therefore be expected to reduce the establishment or expression of pro-inflammatory lactylation programs. Direct evidence for reduced H3K18la at specific IL6, TNF, or NLRP3 regulatory regions remains to be established [68]. The two strategies converge on atheroprotection through distinct genomic targeting of H3K18la

Systemic inhibition of p300/CBP or LDHA, while mechanistically attractive, would exert broad effects given their roles in multiple tissues. We propose that cell-type-selective strategies are required. p300-degrading PROTACs conjugated to macrophage-specific ligands (e.g., mannose or CD163-targeting moieties) could selectively ablate proinflammatory lactylation machinery in plaque-resident immune cells [82]. Biomimetic nanoparticles encapsulating MCT4 or LDHA inhibitors could restrict metabolic suppression to the lesion without disrupting systemic glucose homeostasis [83]. These approaches are predicted to succeed only if pathogenic lactylation memory is maintained by ongoing p300 activity—a prediction that can be tested by assessing H3K18la turnover rates in plaque macrophages ex vivo.

**Fig. 4:**
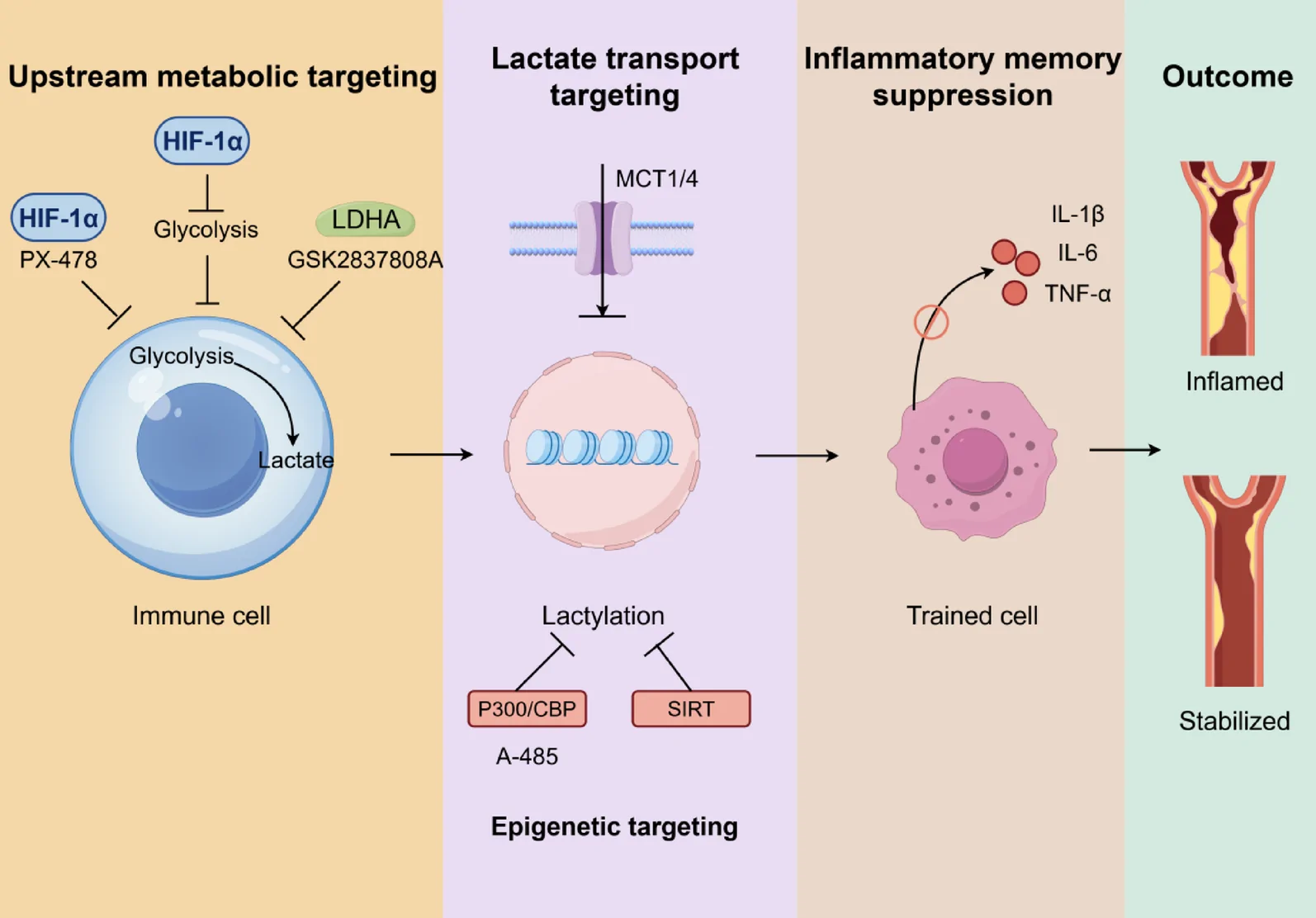
Integrated model for targeting lactate-mediated epigenetic reprogramming in coronary heart disease. **A** Prevention strategy: pharmacological inhibition of p300/CBP (A-485) or LDHA (GSK2837808A) during high-risk windows (post-MI, uncontrolled metabolic stress) to prevent H3K18la memory establishment. **B** Erasure strategy: combination of p300/CBP inhibition with SIRT1 activation (resveratrol, SRT1720) to facilitate active delactylation of consolidated memory marks. **C** Cell-type-selective delivery: macrophage-targeted p300 PROTACs conjugated to mannose or CD163 ligands, and biomimetic nanoparticles encapsulating MCT4 or LDHA inhibitors to restrict metabolic modulation to plaque-resident immune cells. **D** Temporal therapeutic window: the model distinguishes the memory establishment phase (targetable by prevention) from the consolidated memory phase (requiring erasure strategies), emphasizing that indiscriminate lactylation suppression risks abrogating protective programs including exercise-induced atheroprotection and cardiac preconditioning

The temporal dimension is equally critical and suggests three distinct therapeutic strategies. Prevention of memory establishment would employ p300 inhibitors, LDHA inhibitors, or MCT4 blockade during high-risk windows such as post-MI or during uncontrolled metabolic stress, before durable H3K18la deposition has occurred. Suppression of memory expression would use ongoing pharmacological blockade of the glycolytic–lactylation axis to prevent transcriptional output from already-poised loci, reducing inflammatory cytokine production without necessarily removing the underlying mark. Erasure of consolidated memory would require active removal of existing H3K18la marks, for example via sirtuin activation (SIRT1 or SIRT3) or targeted HDAC1–3 recruitment, and may be necessary in advanced disease where memory has already been established over months to years. We emphasize that pharmacological inhibitors of lactate production or lactylation deposition (LDHA inhibitors, MCT4 blockers, p300 inhibitors) are expected to achieve prevention and suppression, but not erasure, of lactylation memory. We further note that indiscriminate lactylation suppression could abrogate protective programs, including Serpina3k lactylation in cardiac ischemia–reperfusion injury [84]. Strategies that selectively reshape lactylation landscapes—modulating lactate flux or enhancing specific delactylation pathways—may offer a more favorable therapeutic window. An integrated model for these therapeutic strategies is proposed in Fig. 4.

## Discussion

We have advanced the hypothesis that histone lactylation, particularly H3K18la, functions as an epigenetic memory code that bridges metabolic reprogramming to sustained innate immune hyperresponsiveness in CHD. The core proposition—that the lactate–p300–H3K18la axis encodes durable, context-dependent epigenetic memory—integrates three converging lines of evidence: the persistence of lactylation relative to classical histone modifications, the demonstration that this mark is causally required for BCG-induced trained immunity [17], and the uniquely pro-glycolytic, HIF-1α-driven microenvironment of the atherosclerotic plaque. We propose the model of “context-dependent lactylation memory”: H3K18la maintains chromatin in a poised structural state, while the specific functional output—proinflammatory or reparative—is decoded by the local cytokine milieu, transcription factor availability, and metabolic flux direction. Critical evidence gaps requiring empirical validation are cataloged in Table 3.

**Table 3:** Critical evidence gaps in the lactylation–trained immunity–CHD axis

Gap	Current status	Priority
Direct evidence from CHD patients	No lactylation mapping from CHD patient circulating monocytes or plaque-resident macrophages	Highest
Cell-type-specific lactylation landscapes	Macrophage studies predominate; data for neutrophils, NK cells, T cells, B cells lacking	High
Site-specific functional divergence	H3K18la dominates literature; roles of H3K9la, H4K12la, and other sites underexplored	High
Temporal dynamics of memory	Persistence demonstrated only in BCG model; decay kinetics in CHD unknown	High
Distinction between enzymatic vs. non-enzymatic lactylation	Relative contributions of p300/CBP-mediated vs. LGSH-mediated pathways in CHD unexplored	High
Therapeutic window definition	Safety and specificity of targeting lactylation machinery undefined	High

### Testable predictions

Our hypothesis generates five specific, falsifiable predictions. Validation of this hypothesis will require complementary approaches in both human cohorts and mouse models. Human studies are essential to establish clinical correlation between H3K18la levels and residual inflammatory risk, while mouse models are required to establish causality through genetic and pharmacological interventions. Bone marrow transplantation experiments can further dissect the relative contributions of central (HSPC) versus peripheral (tissue macrophage) lactylation memory. The five predictions below are structured to be testable across these complementary systems.

First, we predict that H3K18la levels in circulating monocytes from CHD patients will be elevated relative to age-matched controls and will correlate positively with plasma inflammatory biomarkers (hsCRP, IL-6) and negatively with efferocytosis capacity ex vivo. Second, we predict that in atherosclerosis-prone mouse models (Ldlr⁻/⁻ or Apoe⁻/⁻), pulse-chase isotope labeling will demonstrate that H3K18la marks deposited during a defined period of hyperglycemia or oxLDL exposure persist at immune gene loci for at least 4–8 weeks following restoration of normoglycemia or normolipidemia—substantially outlasting the initiating metabolic stimulus. Third, we predict that pharmacological inhibition of p300 during the training phase will prevent the establishment of lactylation-dependent inflammatory memory, whereas p300 inhibition after memory consolidation will require concurrent sirtuin activation to facilitate active erasure. Fourth, we predict that single-cell CUT&Tag profiling of human carotid endarterectomy specimens will reveal enrichment of H3K18la at NLRP3 inflammasome, p38 MAPK pathway, and proinflammatory cytokine gene loci specifically in Trem2hi lipid-associated macrophage subsets, and that this enrichment will be spatially graded with plaque hypoxia as measured by pimonidazole adduct immunohistochemistry. Fifth, we predict that the non-enzymatic LGSH-mediated lactylation pathway predominates in the severely hypoxic plaque core (where severe metabolic stress may constrain enzymatic p300/ -mediated lactylation), whereas enzymatic p300/CBP-mediated lactylation predominates in the relatively oxygenated plaque shoulder region. These predictions are experimentally tractable with current technologies and collectively define a roadmap for validating or refuting the core hypothesis.

### Alternative interpretations and limitations

Several important limitations and alternative interpretations must be considered. First, the proposed role of lactylation as an epigenetic memory code in CHD is currently inferred from indirect evidence—primarily BCG vaccination models, in vitro studies, and animal experiments—and whether lactylation represents a causal driver of inflammatory memory or a downstream reflection of broader metabolic reprogramming remains unresolved. It is possible that H3K18la is a correlative epigenetic mark rather than a causal mediator; distinguishing these alternatives requires the specific loss-of-function experiments outlined above.

Second, the relative contribution of lactylation compared with other epigenetic modifications—particularly H3K27ac and H3K4me3—has not been systematically defined. Trained immunity involves a coordinated epigenetic network, and lactylation may function as one component within a multi-layered regulatory system. Our hypothesis explicitly acknowledges this by positioning lactylation as a critical node rather than the sole driver. If parallel epigenetic marks prove sufficient to sustain trained immunity in the absence of lactylation, the therapeutic relevance of targeting this axis would be diminished.

Furthermore, recent work by Cowley et al. demonstrated that DNA sequence features, particularly CpG dinucleotide density, orchestrate a self-reinforcing epigenetic state involving DNA demethylation, methylation-sensitive transcription factor binding, and sustained H2A.Z incorporation that confers persistent chromatin accessibility at inflammatory gene loci across cellular generations [85]. This finding suggests that histone lactylation may operate atop a DNA-sequence-encoded memory substrate, and the relative contribution of histone-level versus DNA-level mechanisms to the durability of innate immune memory in CHD remains undefined.

Third, the context-dependent duality of lactylation represents both a constraint on the hypothesis and a source of explanatory power. The same H3K18la modification can promote reparative programs in MCT4-deficient macrophages [56] or pathogenic ferroptosis in EP300-overexpressing macrophages [58]. We interpret this duality as evidence that lactylation functions as a permissive—rather than deterministic—chromatin state, which is consistent with our model but also limits its predictive specificity. Resolving the factors that determine functional output at individual genomic loci (reader protein identity, combinatorial modification context, transcription factor co-occupancy) is a priority for refining the hypothesis.

Fourth, the temporal dynamics of lactylation-mediated memory under chronic endogenous stimuli remain undefined. Persistence in the BCG vaccination model [17] may not extrapolate to the sustained, low-grade metabolic stress characteristic of CHD, where continuous stimulus exposure could obscure the distinction between ongoing modification and true memory. Longitudinal studies incorporating stimulus withdrawal protocols are essential.

Fifth, the therapeutic window for selectively targeting pathogenic versus protective lactylation programs has not been established, and the systemic consequences of modulating a pathway with broad physiological roles in tissue repair, stem cell maintenance, and exercise adaptation are unknown. Cell-type-restricted delivery strategies, while promising, remain in preclinical development.

### Bridging trained immunity and residual inflammatory risk

The concept of trained immunity was originally formulated to explain how innate immune cells acquire memory-like properties in response to infectious challenges. Our hypothesis extends this framework by proposing that endogenous metabolic signals—specifically, sustained lactate production in the plaque microenvironment—can hijack the same epigenetic machinery to encode maladaptive innate immune memory. This extension offers a mechanistic account of residual inflammatory risk: the plaque cellular compartment retains an epigenetic memory of past metabolic insults that is structurally encoded in the lactylation landscape and functionally decoded by ongoing microenvironmental cues. If validated, this model would suggest that residual risk is not simply a function of subclinical inflammation, but reflects a fundamental alteration in the chromatin state of plaque-resident and bone marrow-derived immune cells that is not addressed by current lipid-lowering or anti-cytokine therapies.

The context-dependent duality of lactylation, rather than weakening its relevance to CHD, offers a coherent explanation for the chronic, non-resolving character of the disease. In the plaque microenvironment, sustained HIF-1α-driven glycolysis continuously generates lactate, the obligatory substrate for H3K18la deposition [16, 37, 38]. The functional outcome of this deposition is determined by the cellular context: in macrophages with intact lactate efflux and EP300 expression, H3K18la drives pro-inflammatory and ferroptotic gene programs [57, 58], while in MCT4-deficient macrophages, intracellular lactate retention drives H3K18la at reparative gene loci [56]. The duality of lactylation therefore does not contradict its proposed pathogenic role in CHD; it defines the cellular conditions under which pathogenic versus protective outcomes manifest and imposes constraints on therapeutic targeting.

Three features distinguish CHD from other inflammatory conditions and make it a paradigmatic context for testing the lactylation memory hypothesis. First, the atherosclerotic plaque creates a uniquely hypoxic and glycolytic tissue microenvironment that generates sustained, high concentrations of lactate—the obligatory substrate for H3K18la—at levels that drive chronic histone lactylation [16, 43, 46]. Second, while individual plaque macrophages are short-lived and continuously replenished from circulating monocytes, the durability of trained immunity resides in the bone marrow HSPC compartment, where cardiovascular risk factors induce lasting epigenetic reprogramming that biases myelopoiesis toward hyperinflammatory progeny for months to years [7, 8, 10]. This central memory continuously feeds trained monocytes into the plaque, where the local glycolytic microenvironment reinforces peripheral memory through sustained H3K18la deposition. Third, CHD is distinguished by the clinical phenomenon of residual inflammatory risk: recurrent cardiovascular events despite optimal lipid control [1, 3, 4], a measurable outcome that demands a mechanistic explanation beyond lipid-driven inflammation. These features do not preclude a role for lactylation in other diseases, but the convergence of sustained glycolytic substrate supply, durable central HSPC memory, and clinically quantifiable residual risk makes CHD the setting in which the lactylation memory hypothesis can be most rigorously evaluated.

### Priorities for empirical validation

Empirical validation should proceed across three complementary levels. First, human studies should determine whether H3K18la in circulating monocytes and plaque macrophages is associated with inflammatory burden and recurrent cardiovascular events. Second, stimulus-withdrawal and genetic or pharmacological studies in atherosclerosis-prone mouse models should test whether H3K18la persists beyond the initiating metabolic insult and contributes causally to enhanced secondary inflammatory responses. Third, cell-type-resolved and locus-specific profiling should identify the myeloid populations and regulatory regions that carry the proposed memory state and distinguish the relative contributions of enzymatic and non-enzymatic lactylation pathways. Together, these approaches would test the clinical relevance, temporal durability, and causal specificity of the hypothesis without presupposing its validity. Future studies will determine whether the lactate–lactylation axis can be translated from an emerging biological mechanism into a clinically actionable target for mitigating residual inflammatory risk.

## Data Availability

No datasets were generated or analysed during the current study.

## Abbreviations

AARS1/2: Alanyl-tRNA synthetase 1/2
ACS: Acute coronary syndrome
AS: Atherosclerosis
BCG: Bacille Calmette-Guérin
BRG1: Brahma-related gene 1
CBP: CREB-binding protein
CHD: Coronary heart disease
ChIP-seq: Chromatin immunoprecipitation sequencing
CUT&Tag: Cleavage under targets and tagmentation
DAMPs: Damage-associated molecular patterns
EndMT: Endothelial-to-mesenchymal transition
EP300: E1A binding protein p300
GPR81: G protein-coupled receptor 81 (HCAR1)
H3K9la: Histone H3 lysine 9 lactylation
H3K18la: Histone H3 lysine 18 lactylation
H3K27ac: Histone H3 lysine 27 acetylation
H3K4me3: Histone H3 lysine 4 trimethylation
H4K12la: Histone H4 lysine 12 lactylation
HCAR1: Hydroxycarboxylic acid receptor 1 (GPR81)
HDAC: Histone deacetylase
HIF-1α: Hypoxia-inducible factor-1α
HK2: Hexokinase 2
HRE: Hypoxia response element
HSPC: Hematopoietic stem and progenitor cell
IL: Interleukin
LDHA: Lactate dehydrogenase A
LGSH: Lactoylglutathione
MCT1/4: Monocarboxylate transporter 1/4
MGO: Methylglyoxal
MI: Myocardial infarction
NAD^+^: Nicotinamide adenine dinucleotide
oxLDL: Oxidized low-density lipoprotein
PDK1: Pyruvate dehydrogenase kinase 1
PFK1: Phosphofructokinase-1
PHD: Prolyl hydroxylase
PROTAC: Proteolysis-targeting chimera
SIRT1–3: Sirtuin 1–3
TCA: Tricarboxylic acid
TNF-α: Tumor necrosis factor-α
TRIM: Trained immunity
VSMC: Vascular smooth muscle cell
